# Diagnosis and body mass index effects on hippocampal volumes and neurochemistry in bipolar disorder

**DOI:** 10.1038/tp.2017.42

**Published:** 2017-03-28

**Authors:** D J Bond, L E Silveira, E L MacMillan, I J Torres, D J Lang, W Su, W G Honer, R W Lam, L N Yatham

**Affiliations:** 1 Mood Disorders Centre, Department of Psychiatry, University of British Columbia, Vancouver, BC, Canada; 2 Department of Psychiatry, University of Minnesota Medical School, Minneapolis, MN, USA; 3 Laboratory of Molecular Psychiatry, Centro de Pesquisas Experimentais, Hospital de Clínicas de Porto Alegre and INCT for Translational Medicine, Porto Alegre, Brazil; 4 Department of Medicine (Neurology), University of British Columbia, Vancouver, BC, Canada; 5 Department of Radiology, University of British Columbia, Vancouver, BC, Canada; 6 Department of Psychiatry, University of British Columbia, Vancouver, BC, Canada

## Abstract

We previously reported that higher body mass index (BMI) was associated with greater hippocampal glutamate+glutamine in people with bipolar disorder (BD), but not in non-BD healthy comparator subjects (HSs). In the current report, we extend these findings by examining the impact of BD diagnosis and BMI on hippocampal volumes and the concentrations of several additional neurochemicals in 57 early-stage BD patients and 31 HSs. Using 3-T magnetic resonance imaging and magnetic resonance spectroscopy, we measured bilateral hippocampal volumes and the hippocampal concentrations of four neurochemicals relevant to BD: *N*-acetylaspartate+*N*-acteylaspartylglutamate (tNAA), creatine+phosphocreatine (Cre), myoinositol (Ins) and glycerophosphocholine+phosphatidylcholine (Cho). We used multivariate factorial analysis of covariance to investigate the impact of diagnosis (patient vs HS) and BMI category (normal weight vs overweight/obese) on these variables. We found a main effect of diagnosis on hippocampal volumes, with patients having smaller hippocampi than HSs. There was no association between BMI and hippocampal volumes. We found diagnosis and BMI effects on hippocampal neurochemistry, with patients having lower Cre, Ins and Cho, and overweight/obese subjects having higher levels of these chemicals. In patient-only models that controlled for clinical and treatment variables, we detected an additional association between higher BMI and lower tNAA that was absent in HSs. To our knowledge, this was the first study to investigate the relative contributions of BD diagnosis and BMI to hippocampal volumes, and only the second to investigate their contributions to hippocampal chemistry. It provides further evidence that diagnosis and elevated BMI both impact limbic brain areas relevant to BD.

## Introduction

Over two-thirds of Americans are overweight (33%) or obese (39%).^1^ Obesity is one of the strongest risk factors for developing diabetes, hypertension, heart disease, stroke and cancer, and it is the second-leading cause of excess morbidity and mortality in the United States after smoking.^2, 3, 4^ Patients with bipolar disorder (BD) are even more likely to be obese than the overall population—in fact, obesity rates are over 60% greater in BD patients.^5^ Not surprisingly, they also suffer more metabolic illnesses, including 25% higher rates of hypertension and 200–300% higher rates of diabetes.^6, 7, 8, 9, 10, 11, 12^ Much of this excess medical burden is directly attributable to obesity, as shown by a population-based study that reported that obese BD patients had 35–96% greater rates of hypertension, arteriosclerosis and myocardial infarction than normal-weight patients.^5^

Animal models demonstrate that the health consequences of obesity are caused by body mass index (BMI)-related changes in adipose tissue physiology and in the blood levels of biomarkers made by adipose tissue, such as inflammatory cytokines, adipokines, and pro-oxidative and thrombotic factors.^13, 14, 15, 16^ This culminates in multiorgan endothelial dysfunction, the final common pathway for obesity-related medical complications.^17^ Obesity is associated with similar biomarker alterations in humans, and many of these biomarkers cross the blood–brain barrier.^18, 19, 20, 21, 22, 23^ Thus, it stands to reason that the human brain will be susceptible to obesity-related pathology. Supporting this hypothesis, BMI-related endothelial damage has been demonstrated in the brain, and age-related brain volume reductions are more pronounced in primates and humans with higher BMIs.^17, 24, 25, 26^ Diet-induced obesity in a mouse model of Alzheimer's disease resulted in increased β-amyloid production, whereas humans who are obese in midlife have a twofold increased risk of developing dementia.^27, 28^ Obesity is also a risk factor for other brain diseases including multiple sclerosis and Parkinson's disease.^29, 30, 31, 32^

These facts, coupled with the high rate of obesity in BD and evidence that obese patients have a more severe psychiatric illness course than normal-weight patients,^5, 33, 34, 35, 36^ led us and others to investigate the impact of elevated BMI on brain illness severity in BD. Using magnetic resonance imaging (MRI), we found BMI-related gray and white matter (GM and WM) volume reductions and decreased WM integrity in limbic brain areas in patients, but not non-BD comparison subjects.^37, 38, 39^ The volume reductions were particularly pronounced in the temporal lobes, especially the right temporal lobe. Our group also demonstrated BMI-related increases in hippocampal glutamate+glutamine in patients.^40^ Elevated glutamate+glutamine is the most consistently reported neurochemical abnormality in BD.^41^ These findings thus suggest that brain areas vulnerable in BD experience further BMI-related damage, so that higher BMI exacerbates the neuropathology of BD.

In the current report, we extend these investigations by examining the impact of elevated BMI on hippocampal volumes and the concentrations of several additional neurochemicals in early-stage BD patients. To evaluate the specificity of our findings to BD, we also included a comparison group of non-BD healthy subjects (HSs). The hippocampus plays important roles in reward processing and emotional memory. A recent meta-analysis reported hippocampal volume reductions in BD, especially in younger patients.^42, 43, 44^ The hippocampus also appears to be particularly sensitive to obesity-induced damage, even relative to other brain areas.^45^ The neurochemicals we measured are all relevant to BD and include *N*-acetylaspartate, a marker of neuron and myelin function; creatine, which plays a key role in cellular energetics; myoinositol, a second messenger important in phosphoinositol intracellular signalling cascades; and phosphatidylcholine/glycerophosphocholine, which are important in neuronal and glial cell membrane biosynthesis. We hypothesized that BD diagnosis and higher BMI would both be associated with smaller hippocampal volumes and neurochemical abnormalities, and that the impact of BMI would be greater in patients than HS.

## Materials and methods

### Systematic Treatment Optimization Program for Early Mania

The Systematic Treatment Optimization Program for Early Mania is a study of clinical outcomes, brain morphology and neurochemistry following the first manic episode in patients with BD.^46^ It is based at the University of British Columbia Mood Disorders Centre in Vancouver, Canada. In brief, BD patients aged 14–35 years who experienced their first DSM-IV-TR-defined manic/mixed episode in the preceding 3 months were recruited from the Mood Disorders Clinical Research Inpatient Unit, the Mood Disorders Outpatient Program and affiliated community sites. To capture the full spectrum of BD patients seen in clinical practice, patients with comorbid psychiatric and substance use disorders could be enrolled so long as the primary diagnosis was BD. HSs aged 14–35 years with no history of psychiatric illness were recruited from the greater Vancouver metropolitan area through advertisements and online forums such as Craigslist. Patients and HSs with histories of head trauma with the loss of consciousness were excluded. The University of British Columbia Clinical Research Ethics Board approved the Systematic Treatment Optimization Program for Early Mania and written informed consent was obtained prior to any study procedures taking place.

### Clinical assessment

At enrolment, the diagnoses of BD and first manic/mixed episode were made based on a comprehensive interview with an academic research psychiatrist, and confirmed with the Mini-International Neuropsychiatric Interview.^47^ HSs were also administered the Mini-International Neuropsychiatric Interview, and were enrolled if they had no personal or family history (in first-degree relatives) of psychiatric illness. Sociodemographic and clinical data were collected using a standardized protocol. Mood and psychotic symptoms were quantified in patients with the Young Mania Rating Scale, the Montgomery–Asberg Depression Rating Scale, and the Positive and Negative Syndrome Scale. Patients received treatment for BD according to clinical practice guidelines from the Canadian Network for Mood and Anxiety Treatments.^48, 49^ Subjects were weighed in a non-fasting state in light clothing with footwear removed. BMI was calculated as weight (kg)/height (m).^2^ Underweight was defined as BMI<18.50, normal weight as BMI=18.50–24.99, overweight as BMI=25.00–29.99 and obesity as BMI⩾30.00. ^50^

### MRI/MRS protocols and data extraction

T1-weighted MR images were acquired with a Philips Achieva 3-Tesla scanner (Best, The Netherlands), typically on the same day as clinical assessment. A three-dimensional axial inversion recovery-weighted spoiled gradient recalled sequence with the following parameters was used: field of view=25.6 cm, matrix=256 × 200, isotropic voxels (1 × 1 × 1 mm^3^), autoshim, repetition time/echo time=7.6/3.5 ms, transmit/receive head coil, flip angle=8°, SENSE=0 and 1 mm-thick contiguous 180 slices of the whole brain. Hippocampal volumes were extracted using Freesurfer v5.3 (http://surfer.nmr.mgh.harvard.edu) subcortical segmentation. The GM, WM and cerebrospinal fluid (CSF) composition of the magnetic resonance spectroscopy (MRS) voxel was determined with the FSL v4.1.9 FAST tool (FMRIB Software Library; www.fmrib.ox.ac.uk/fsl/).^51, 52^

Proton-MRS (^1^H-MRS) signals were acquired with the Philips 3-T unit. T2-weighted coronal, sagittal and axial images were used to prescribe two 30 × 15 × 15 mm voxels in the left and right hippocampi. Each voxel was placed with its long axis angled along the hippocampus. Its position in the medial/lateral and superior/inferior directions was adjusted to include the maximum amount of hippocampus and avoid CSF. Screen captures of the slice positioning and anatomical landmarks for voxel placement were acquired for each subject. These were referenced by the scanning technologist to ensure consistency of voxel placement across subjects. Voxel placement and tissue composition are shown in Supplementary Figure S1a. Point-resolved spectroscopy with echo time=35 ms, repetition time=2000 ms and water suppression via chemical shift selective suppression excitation were used to acquire the spectra. One hundred twenty-eight water-suppressed and 16 non-water-suppressed averages were acquired from each voxel. The non-water-suppressed signals were used for eddy current correction and to reference metabolite signals.

Hippocampal concentrations of four metabolites—*N*-acetylasparate+*N*-acetylaspartylglutamate (tNAA), creatine+phosphocreatine (Cre), myoinositol (Ins), and glycerophosphocholine+phosphatidylcholine (Cho)—were extracted using LCModel v. 6.3^53^ and normalized to the unsuppressed water spectrum. Sample MRS spectra from a patient and a HS are displayed in Supplementary Figure S1b. Metabolite-to-water ratios were converted to institutional absolute concentrations in millimolar units by correcting for the mean water concentration of the voxel (taking into account the fractions of GM, WM and CSF in the voxel and their respective water concentrations), while accounting for water and metabolite signal T1 and T2 relaxations (Supplementary Materials and Methods). Values for water and metabolite T1 and T2 relaxation times were taken from the literature.^54, 55^ The tissue composition of the MRS voxel and measures of MRS data quality, including signal-to-noise ratio and full width at half maximum, is shown in Supplementary Table S1.

### Data analyses and statistics

Statistical analyses were carried out using IBM SPSS Statistics for Windows 22.0 (SPSS, Chicago, IL, USA). Comparisons were two-tailed, with a significance level of *α*=0.05. We examined sociodemographic and clinical variables with *t*-tests, *χ*^2^-tests and Fisher's exact test as appropriate. Our primary and secondary analyses are specified below. We did not otherwise correct for multiple comparisons. Statistical assumptions appropriate to the tests, including normality of dependent variables and homogeneity of variances, were met.

#### Primary analyses

Our main objective was to investigate the association of diagnosis, BMI category, and their interaction with hippocampal volumes and neurochemistry. To determine whether diagnosis (BD patient vs HS) and BMI category (normal weight vs overweight/obese) were associated with hippocampal volumes, we created a factorial multivariate analysis of covariance (MANCOVA) model. Diagnosis and BMI category were factors, and left and right hippocampal volumes were the dependent variables. To determine whether the impact of BMI differed in patients and HSs, a diagnosis × BMI category interaction term was also included. Age and years of education were covariates. A positive result for diagnosis, BMI category, or the interaction was followed up with analyses of covariance including these three factors to assess left and right hippocampal volumes separately. Because some published reports on hippocampal volumes in BD adjusted for total intracranial volume (TICV; total GM+total WM+CSF), whereas others did not (for an in-depth review see Otten and Meeter^44^), we carried out these analyses with and without adjusting for TICV.

To determine whether diagnosis, BMI category and their interaction were associated with overall hippocampal neurochemistry while minimizing multiple comparisons, we created a factorial MANCOVA model. Left and right tNAA, Cre, Ins and Cho were dependent variables, and diagnosis, BMI category and their interaction were factors. Covariates included age and years of education. To ensure that neurochemical findings were independent of diagnosis- or BMI-related differences in hippocampal volumes or the GM/WM/CSF composition of the MRS voxels, these were also covariates. A positive result for diagnosis, BMI or the interaction was followed up with analyses of covariance including these factors to assess each metabolite separately. We excluded poor-quality data for individual metabolite values by (1) multiplying the %s.d. error from LCModel by the corresponding absolute concentration (mM) to obtain the absolute error estimate, and (2) rejecting data when the absolute error estimate was >30% of the median metabolite concentration across all subjects. Compared with the conventional threshold of rejecting data with %s.d. error estimates >20%, this method ensures that there is less bias towards rejecting low-concentration metabolite values.^56^

#### Secondary outcomes

We also constructed multivariate multiple regression models separately in patients and HSs to investigate the impact of the continuous variable of BMI on hippocampal volumes and neurochemistry in the two groups independently. In patients, this allowed us to assess the impact of BMI on these measures while adjusting for confounding clinical and medication variables. In HSs, it was an important confirmatory analysis as only *N*=6 HSs were overweight/obese, giving us relatively low statistical power to detect BMI category effects in HSs. The regressions, in contrast, allowed us to look for a BMI effect over the entire continuous range of values. In the volumetric regressions, left and right hippocampal volumes were the dependent variables. Predictors for patients included BMI; Young Mania Rating Scale, Montgomery–Asberg Depression Rating Scale, and Positive and Negative Syndrome Scale scores; treatment with mood stabilizers and second-generation antipsychotics; age; and years of education. For HSs, they included BMI, age and years of education. As with the factorial models, these were carried out with and without controlling for TICV. In the neurochemical multivariate regressions, left and right tNAA, Cre, Ins and Cho were the dependent variables. Predictors for patients included Young Mania Rating Scale and Montgomery–Asberg Depression Rating Scale scores, treatment with mood stabilizers and second-generation antipsychotics, BMI, age, years of education, hippocampal volumes, and the composition of the MRS voxels. Predictors for HSs included BMI, age, years of education, hippocampal volumes and the composition of the MRS voxels.

## Results

### Participants

Fifty-seven of 71 consecutively enrolled patients and 31 of 42 HSs enrolled in the Systematic Treatment Optimization Program for Early Mania had baseline BMI and MR data, and were included in the volumetric analyses. Participants with missing data had declined to be weighed or to undergo the MR procedure. Three patients and 1 HS were excluded from the neurochemical analyses for having artefacts in their MRS data or having an absolute error estimate >30% of the median metabolite concentration for one or more metabolites, leaving 54 patients and 30 HS for these.

Patients and HSs were well matched on sociodemographic characteristics, except that HSs had more years of education (Table 1). In keeping with their early illness stage, patients and HSs had similar mean BMIs and proportions with normal weight, overweight and obesity. Ninety-three percent of patients were treated with mood stabilizers and/or second-generation antipsychotics (Table 2). Seventy-five percent met a stringent definition of euthymia (Montgomery–Asberg Depression Rating Scale<12 and Young Mania Rating Scale<12), 14% had subsyndromal depressive or hypomanic symptoms and 11% were depressed or manic.

### Diagnosis, BMI and hippocampal volumes

#### Factorial MANCOVA

**Figure 1:**
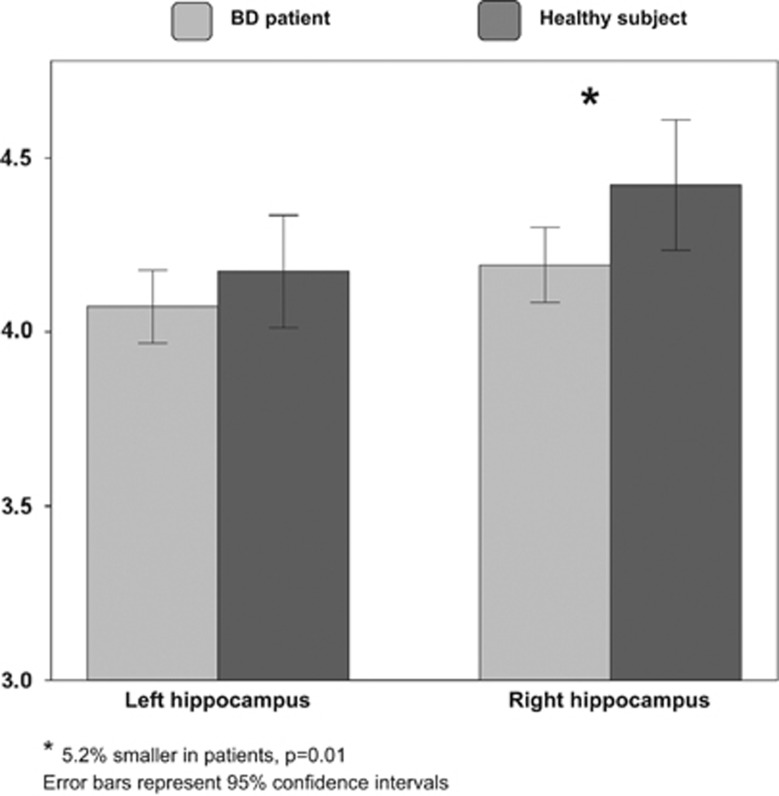
Left and right hippocampal volumes in bipolar disorder (BD) patients and healthy subjects.

The factorial MANCOVA model without adjustment for TICV detected a significant main effect of diagnosis on hippocampal volumes (F[2,81]=6.771, *P*=0.002). There was no main effect of BMI category (F[2,81]=1.741, *P*=0.18) and no diagnosis × BMI interaction (F[2,81]=1.729, *P*=0.18). Follow-up factorial analyses of covariance showed that right hippocampal volume was significantly smaller in patients than HSs (left: 2.4% smaller, 4.07 [0.39] vs 4.17 [0.44] ml, F[1]=1.190, df=1, *P*=0.28; right: 5.2% smaller; 4.19 [0.41] vs 4.42 [0.51] ml; F[1]=6.757, *P*=0.01) (Figure 1). The results were unchanged when TICV was included as a covariate.

#### Linear regression models

In keeping with the results of the MANCOVA, the linear regressions showed that BMI did not predict hippocampal volumes in patients or HSs (all *P*>0.34). Except for TICV, sociodemographic variables in patients and HSs did not predict hippocampal volumes. Clinical and treatment variables in patients also did not predict hippocampal volumes.

### Diagnosis, BMI and hippocampal neurochemistry

#### Factorial MANCOVA

Factorial MANCOVA detected a significant main effect of BMI category on overall hippocampal neurochemistry (F[8,65]=2.108, *P*<0.05). There was no main effect of diagnosis (F[8,65]=1.155, *P*=0.34) and no diagnosis × BMI interaction (F[8,65]=0.827, *P*=0.58). In the follow-up analyses of covariance, both BMI category and diagnosis effects were seen for right Cre, right Ins and right Cho. Each of these metabolites had higher concentrations in overweight/obese than normal-weight subjects (Cre: 3.0% higher, 8.69 [1.04] vs 8.44 [1.24] mM, F[1]=6.203, *P*=0.02; Ins: 14.2% higher, 7.73 [2.36] vs 6.77 [1.93] mM, F[1]=7.201, *P*=0.009; Cho: 3.2% higher, 2.60 [0.36] vs 2.52 [0.44] mM, F[1]=3.907, *P*=0.05), and lower concentrations in patients than HSs (Cre: 3.7% lower, 8.40 [1.20] vs 8.72 [1.14] mM, F[1]=6.191, *P*=0.02; Ins: 2.1% lower, 7.01 [2.02] vs 7.16 [2.27] mM, F[1]=4.594, *P*=0.04; Cho: 3.8% lower, 2.51 [0.46] vs 2.61 [0.44] mM, F[1]=4.138, *P*=0.05).

#### Linear regression models

**Figure 2:**
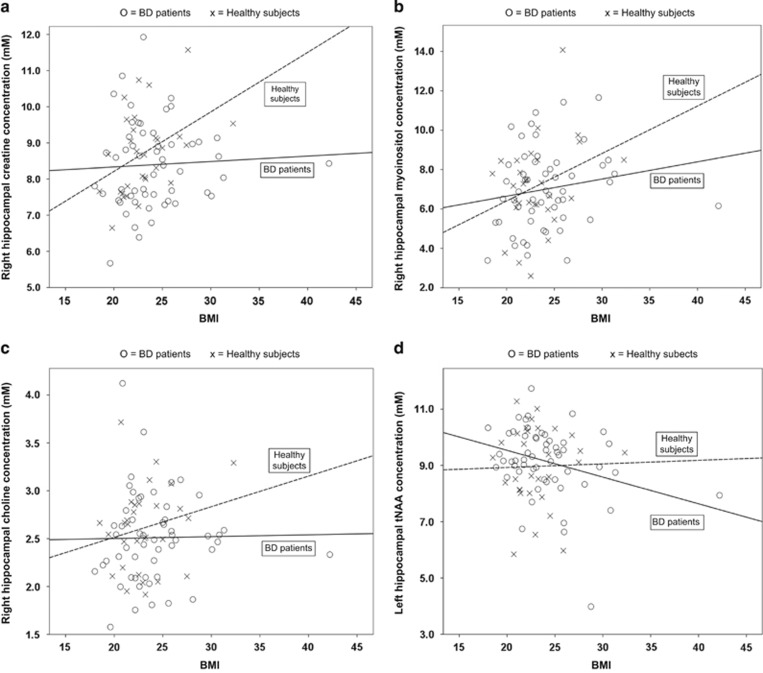
Relationship between body mass index (BMI) and (**a**) right hippocampal creatine+phosphocreatine, (**b**) right hippocampal myoinositol, (**c**) right hippocampal glycerophosphocholine+phosphatidylcholine, and (**d**) left hippocampal *N*-acetylaspartate+*N*-acteylaspartylglutamate (tNAA) in bipolar disorder (BD) patients and healthy subjects.

Although diagnosis × BMI category interactions were not detected in the above models, Figures 2a–c suggest that the association between higher BMI and higher neurometabolite levels was more pronounced in HSs than patients. This was borne out by the linear regressions, which showed significant relationships between greater BMI and higher right Cre and Ins in HSs (Cre: F[1]=6.030, *P*=0.02 (Figure 2a); Ins: F[1]=4.832, *P*=0.04 (Figure 2b)), but not patients (Cre: F[1]=0.056, *P*=0.81; Ins: F[1]=1,777, *P*=0.19). BMI was not significantly associated with right Cho in HSs or patients (Figure 2c). Interestingly, controlling for clinical/treatment variables in patients revealed a relationship between higher BMI and lower left tNAA (F[1]=5.567, *P*=0.02; Figure 2d).

## Discussion

To our knowledge, this was the first study to investigate the relative contributions of BD diagnosis and elevated BMI to hippocampal volumes, and only the second (after our glutamate+glutamine study) to investigate their contributions to hippocampal chemistry. We found a diagnosis effect on hippocampal volumes, with patients having smaller hippocampi than HSs, a finding that was most pronounced in the right hippocampus. There was no association between higher BMI and hippocampal volumes. We detected diagnosis- and BMI-related differences in hippocampal chemistry, with patients having lower right Cre, MI and Cho, and overweight/obese subjects having higher levels of these chemicals.

Our patient-only models allowed us to control for clinical and treatment factors, and thus enabled us to detect an additional relationship between higher BMI and lower left tNAA in patients that was not apparent in the primary models. One possible explanation for the absence of this finding in the primary models this is a normalizing effect of mood-stabilizing medications on tNAA, which has been reported in previous studies.^57, 58, 59, 60^ By this logic, the primary models (patients+HSs) did not find a BMI effect on tNAA because the effect in patients was masked by pharmacotherapy, and there was no effect in HS. The patient-only models, by adjusting for the impact of medications, were able to detect the BMI effect. Thus, increased BMI was associated with altered neurochemistry in patients and HSs, though affecting different neurochemicals in each group.

Together with our previous MRI and MRS studies, the current results demonstrate that BD diagnosis and higher BMI both impact brain areas relevant to BD. We previously reported that BD patients, but not HS, had BMI-related reductions in total brain WM and total temporal lobe volume. ^37^ GM was relatively spared, but there was a single focus of volume reduction in the right temporal lobe, suggesting that this area may be particularly vulnerable to BMI-related changes.^38^ Moreover, we also found a BMI-related increase in hippocampal glutamate+glutamine in patients.^40^ The consistency of the BMI effect across measures was striking. It is interesting that our current results differ from our previous findings in one important respect. In the previous studies, BMI-related limbic brain changes were consistently more pronounced in patients than HSs. The current study produced mixed results: higher BMI in patients predicted reduced tNAA, higher BMI in HSs predicted greater Cre and Ins. This suggests that the relationship between BMI and the brain is complex, differing not only between patients and HSs, but also between neurobiological measures.

The mechanisms underlying the relationship between higher BMI and structural and chemical brain changes in BD are unknown. BMI-related alterations in inflammatory cytokines, adipokines and other biomarkers may play a role. Peripheral inflammation affects brain function via the presence of transport mechanisms for cytokines across the blood–brain barrier, as well as cytokine receptors on the blood–brain barrier and the vagus nerve.^61, 62^ Supporting this hypothesis, experimentally induced obesity causes brain inflammation in preclinical models.^63^ Neuroinflammation is increasingly recognized to be important to the etiology of BD, as shown by a recent positron emission tomography study that found hippocampal inflammation in BD patients.^64^ In addition, adipokines such as leptin and adiponectin have receptors in limbic brain areas including the hippocampus and the ventral striatum.^65, 66, 67, 68, 69, 70^ Low serum leptin is associated with reduced nucleus accumbens activity, and low serum adiponectin with hippocampal excitotoxicity.^68, 71, 72, 73^ However, it is also important to remember that the cross-sectional design of the current study means we cannot determine the direction of the link between BMI and the hippocampus. Reverse causality cannot be excluded, particularly in light of the fact that impairment in hippocampal-dependent cognitive functions predicts weight gain.^74^

The hippocampus plays a key role in the pathophysiology of BD. It has reciprocal connections, directly or via the entorhinal cortex, with brain areas involved in the generation and modulation of emotions, including the medial prefrontal cortex, amygdala and ventral striatum.^75^ Current models of BD conceptualize the hippocampus as a crucial node in a prefrontal–hippocampal–amygdala emotion-processing circuit that is underactive in BD.^76^ Our volumetric results are in keeping with meta-analyses of neuroimaging studies that reported reduced hippocampal volumes in BD.^44, 77^ It is more difficult to place the lack of association between BMI and hippocampal volumes into context, as the relationship between BMI and the hippocampus has been less well studied. All of the investigations so far have been in pediatric or older adult samples and have produced inconsistent results. ^78, 79, 80, 81, 82^

The neurochemicals we studied are highly relevant to the pathophysiology of BD. NAA is the second most abundant amino acid in the brain after glutamate. It is produced exclusively in neuronal mitochondria and is considered to be a marker of neuron and myelin function and mitochondrial health.^83, 84, 85^ Reduced NAA is one of the most consistent neurochemical findings in BD.^85^ Cre and PCre exist in equilibrium in a reaction catalyzed by creatine kinase. They are present at high concentrations in cells with large but fluctuating energy demands, such as neurons, where PCre serves as a phosphate donor to synthesize ATP from ADP during periods of high ATP utilization.^86^ A large fraction of creatine kinase is mitochondrial, and alterations in Cre+PCre levels may reflect mitochondrial dysfunction, which is believed to be important to the etiology of BD.^87^ Inositols, mainly Ins, are precursors to myelin and neuronal cell membrane phospholipids such as phosphatidylinositol. They are also components of the phosphoinositol second-messenger system, which regulates intracellular calcium levels and neurotransmitter release.^85, 88^ Changes in inositol levels could thus reflect abnormalities in cell membrane biosynthesis or second-messenger cascades involved in cell survival and intracellular trafficking.^89^ Lithium, divalproex and carbamazepine all act on inositol monophosphatase, the final enzyme in Ins synthesis.^90^ Cholines, primarily phosphocholine and glycerophosphocholine, are precursors to phospholipids such as phosphatidylcholine, and to acetylcholine, which is important to memory. Changes in choline levels may reflect changes in cell membrane turnover, which can occur in brain illnesses and during neuroinflammation.^85, 88^

Nonetheless, studies investigating hippocampal neurochemistry in BD have produced mixed results.^91, 92, 93, 94, 95, 96, 97, 98, 99, 100, 101, 102, 103, 104, 105, 106^ The most consistent finding was reduced NAA, which was detected in 7/15 studies.^91, 92, 93, 94, 97, 100, 103^ One study reported increased NAA,^102^ and the remainder found no diagnosis-related differences. None of the five studies that examined Ins found differences between patients and HSs. Increased Cho was detected in 3/13 studies, with the remainder finding no difference between patients and controls.^93, 102, 104^ Finally, 2/8 studies reported decreased Cre,^97, 100^ 1 reported increased Cre^102^ and the remainder found no diagnosis-related differences. The heterogeneity among study findings may be in part related to small sample sizes, which were *N*⩽40 in 11/16 studies, and differences in illness stage, mood state or pharmacotherapy. Our findings suggest that between-study differences in BMI may also be an important contributing factor.

Strengths of the current study include the following: (1) our well-characterized BD patients and HSs; (2) our first-episode sample, which minimized confounding factors such as variable illness durations, multiple medication trials and high comorbidity rates; and (3) our rigorous quantification of hippocampal volumes and absolute metabolite concentrations. Its limitations include the following: (1) its cross-sectional design; (2) that we did not gather information on factors that might modulate the relationship between BMI and the brain, such as diet, physical activity and smoking, (3) the possibility that random differences in voxel placement created noise in the data, and (4) the limited ability of 3 T MRS to distinguish between closely related molecules, so that, for example, we could not discern low-energy Cre from high-energy PCre.

In conclusion, this report adds to the accumulating evidence that BD diagnosis and elevated BMI both impact brain areas relevant to BD. It also suggests a number of directions for future research. (1) Higher-field-strength MRS (4- T and even 7 -T platforms are now available) would permit better differentiation of closely related neurochemicals and thus allow a more precise quantification of the impact of higher BMI on neurochemistry. (2) Novel MRS techniques such as ^13^C-MRS could provide information on neurochemical pathways and functioning to complement the information on concentrations provided by ^1^H-MRS. (3) Longitudinal studies are urgently needed to determine whether higher BMI and/or increasing BMI over time lead to progression of neurochemical abnormalities in BD. (4) Finally, whether the relationship between BMI and the brain is also important in other psychiatric illnesses with high obesity rates, such as major depressive disorder and schizophrenia, requires investigation.

## Disclaimer

The sponsor had no input into the design or conduct of the study; collection, management, analysis or interpretation of the data; preparation, review or approval of the manuscript; or decision to submit the manuscript for publication.

## Figures and Tables

**Table 1:** Sociodemographic characteristics of BD patients and healthy subjects

	*BD patients (*N*=57)*	*Healthy subjects (*N*=31)*	P*-value*
	*Mean (s.d.)*	*Mean (s.d.)*	
BMI	24.1 (3.9)	23.1 (2.8)	0.24
Age	22.7 (4.5)	22.9 (4.5)	0.84
Years of education	13.9 (2.3)	15.1 (2.6)	0.03

	*Percent (*N*)*	*Percent (*N*)*	
*BMI category*			0.28
Normal weight	64.9% (37)	80.6% (25)	
Overweight	26.3% (15)	16.1% (5)	
Obese	8.8% (5)	3.2% (1)	

*Gender*			0.84
Male	47.4% (27)	45.2% (14)	
Female	52.6% (30)	54.8% (17)	

*Ethnicity (self-reported)*			0.11
Caucasian	78.9% (45)	64.5% (20)	
Asian	17.5% (10)	35.5% (11)	
Other	3.5% (2)	0% (0)	

Abbreviations: BD, bipolar disorder; BMI, body mass index.

**Table 2:** Clinical and treatment characteristics of BD patients

	*Mean (s.d.)*
*Rating scale scores*	
YMRS	3.7 (5.9)
MADRS	6.0 (8.0)
PANSS-positive scale	7.7 (1.5)

Duration of first manic/mixed episode (days)^a^	60.2 (49.8)
Total duration of mood disorder (years; including previous depressions and hypomanias)^a^	2.9 (4.2)

	*Percent (*N*)*
*Mood state*^a^	
Euthymic (MADRS<12 and YMRS<12)	75.0% (42)
Subsyndromal depression (MADRS 12–19)	7.1% (4)
Depressed (MADRS⩾20)	8.9% (5)
Hypomanic (YMRS 12–19)	7.1% (4)
Manic (YMRS⩾20)	1.8% (1)
	
*Pharmacotherapy*	
Mood stabilizer	86.0% (49)
Second-generation antipsychotic	78.9% (45)
Mood stabilizer+antipsychotic	71.9% (41)
No medication	7.0% (4)

Previous depressive episode^a^	53.6% (30)
Previous hypomanic episode^a^	19.6% (11)
	
*Lifetime comorbidity*	
Anxiety disorder^a^	10.7% (6)
Alcohol dependence^b^	3.6% (2)
Drug dependence^b^	8.9% (5)

Abbreviations: BD, bipolar disorder; MADRS, Montgomery–Asberg Depression Rating Scale; PANSS, Positive and Negative Syndrome Scale; YMRS, Young Mania Rating Scale.

a *N*=56; one value missing.

b *N*=55; two values missing.
