# Supplementary material for: Diagnosis and body mass index effects on hippocampal volumes and neurochemistry in bipolar disorder
Source: Transl Psychiatry. 2017 Mar 28;7(3):e1071–. doi: 10.1038/tp.2017.42 (PMC5404613; doi:10.1038/tp.2017.42)
Supplement: Supplementary Information [file tp201742x1.doc]

**Supplementary information**

Figure S1a: Placement of the hippocampal ^1^H-MRS voxel


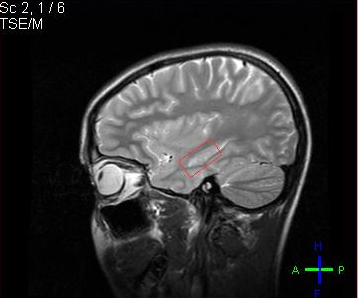


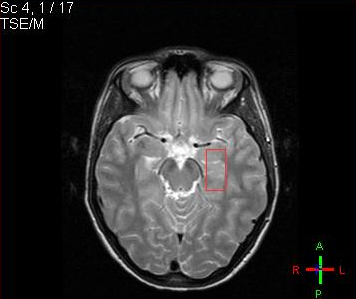


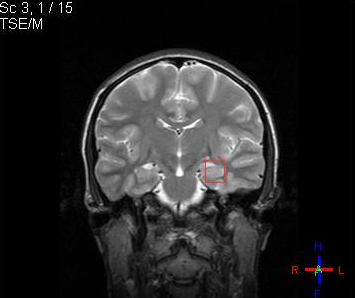


Figure S1b: Sample ^1^H-MRS spectra from a BD patient and a healthy subject


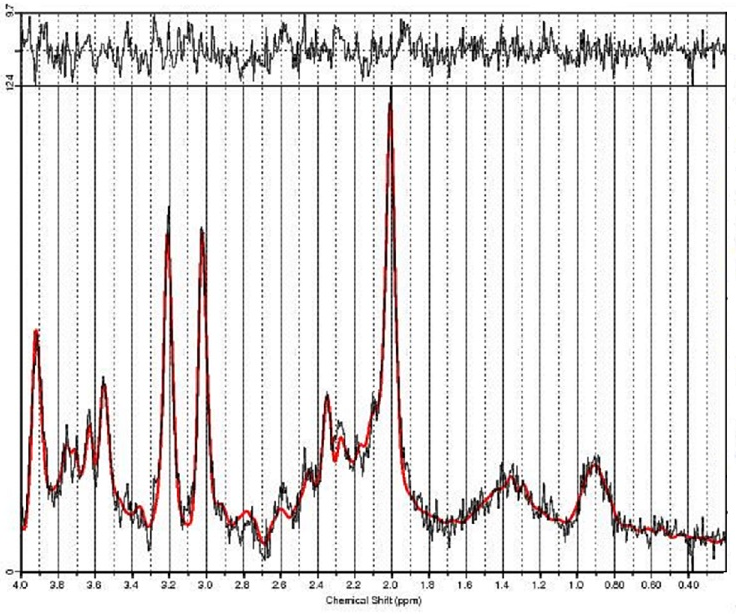


tNAA

Glx

Cre

Cho

Ins


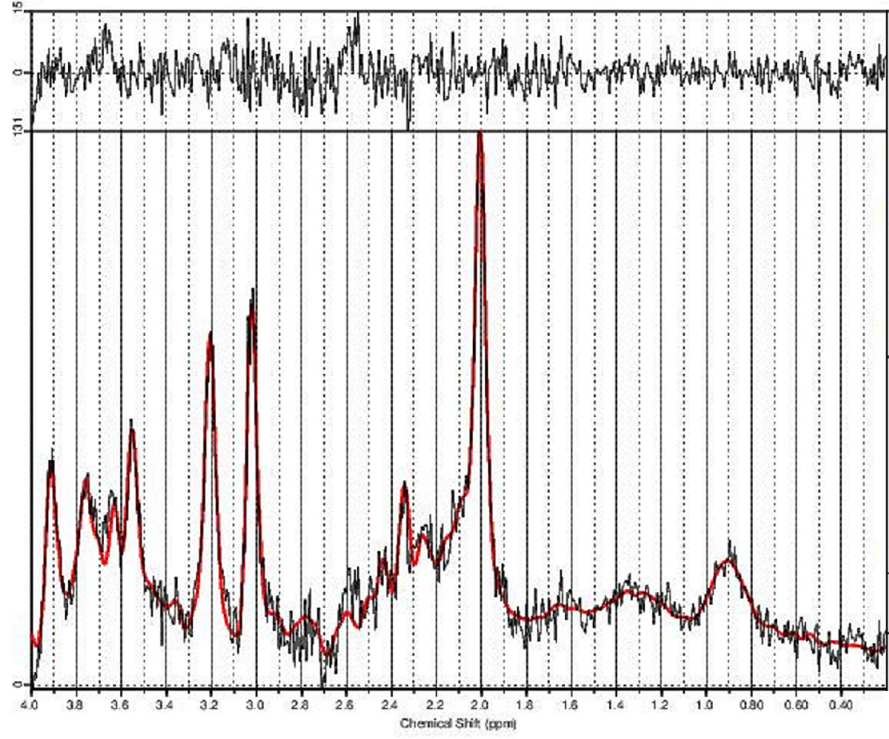


tNAAA

Glx

Cre

Cho

Ins

Cho = phosphatidylcholine+glycerophosphocholine; Cre = creatine+phosphocreatine; Glx = glutamate+glutamine; Ins = myo-inositol; tNAA = N-acetylaspartate+N-acetylaspartylglutamate

Supplementary Materials and Methods

To calculate absolute metabolite concentrations in institutional millimolar units (MET) from the relative water-referenced metabolite concentrations generated by LCModel (MET_LCModel_), the relative concentrations were multiplied by the mean voxel water concentration (WCONC) and a correction factor accounting for water (ATTH2O), and divided by a correction factor accounting for CSF in the voxel:


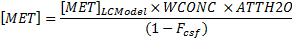


Where:


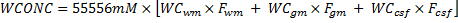


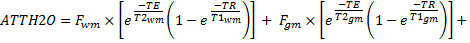


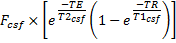


Since out TR was <3.5s and TE was >30ms, we also corrected for metabolite signal T_1_ and T_2_ relaxation during the acquisition by dividing each metabolite’s concentration by ATTMET:


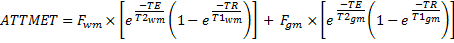


**Key:**

*F_type_* is the volume fraction of each tissue type (eg. if the voxel is composed of 70% WM, *F_wm_* = 0.70)

*TE* is the echo time in seconds

*TR* is the repetition time in seconds

For WCONC and ATTH2O:

*WC_type_* is the percent concentration wet weight of water in the tissue type (eg. CSF is 99% water so *WC_csf_* = 0.99)

*T2_type_* is the T_2_ relaxation time in seconds of water protons in each tissue type

*T1_type_* is the T_1_ relaxation time in seconds of water protons in each tissue type

For ATTMET:

*T2_type_* is the T_2_ relaxation time in seconds of the metabolite in each tissue type

*T1_type_* is the T_1_ relaxation time in seconds of the metabolite in each tissue type

Note: there should not be any metabolites in the CSF so that tissue type is not included.

Table S1: Mean tissue composition of the MRS voxel and MRS data quality measures in BD patients and healthy subjects

|  | **Patients with BD (N=54)** | | **Healthy Subjects (N=30)** | |  |  |  |
| --- | --- | --- | --- | --- | --- | --- | --- |
| **Volumes (mL)** | **Overweight/ Obese (N=19)** | **Normal Weight (N=35)** | **Overweight/ Obese (N=6)** | **Normal Weight (N=24)** | **Diagnosis p-value** | **BMI p-value** | **Diagnosis x BMI p-value** |
|  |  |  |  |  |  |  |  |
|  | **Mean (SD)** | **Mean (SD)** | **Mean (SD)** | **Mean (SD)** |  |  |  |
| **MRS voxel** |  |  |  |  |  |  |  |
| Left voxel GM | 3.79 (0.45) | 3.88 (0.34) | 3.74 (0.33) | 3.87 (0.40) | .80 | .30 | .86 |
| Left voxel WM | 2.74 (0.49) | 2.63 (0.36) | 2.80 (0.38) | 2.65 (0.44) | .71 | .27 | .83 |
| Left voxel CSF | 0.23 (0.09) | 0.24 (0.14) | 0.21 (0.08) | 0.23 (0.08) | .59 | .59 | .85 |
| Right voxel GM | 3.79 (0.47) | 3.67 (0.40) | 3.70 (0.57) | 3.69 (0.35) | .76 | .58 | .62 |
| Right voxel WM | 2.76 (0.47) | 2.87 (0.43) | 2.88 (0.60) | 2.88 (0.37) | .57 | .61 | .63 |
| Right voxel CSF | 0.21 (0.06) | 0.21 (0.13) | 0.18 (0.04) | 0.18 (0.08) | .24 | .93 | .99 |
| **Data quality measures** |  |  |  |  |  |  |  |
| Signal-to-noise ratio (SNR) | 8.39 (2.26) | 7.91 (1.41) | 7.00 (2.19) | 8.19 (1.93) | .26 | .48 | .10 |
| Full width at half maximum (FWHM), Hz | 7.77 (1.46) | 7.60 (1.49) | 7.32 (2.06) | 7.10 (1.44) | .25 | .64 | .95 |

BD = bipolar disorder. FWHM = full width at half-maximum. GM = gray matter. SNR = signal-to-noise ratio. WM = white matter.

The diagnosis x BMI p-value shows the probability that BMI-related changes in hippocampal voxel content and data quality values differ between patients and healthy subjects.
